# More push from your push-off: Joint-level modifications to modulate propulsive forces in old age

**DOI:** 10.1371/journal.pone.0201407

**Published:** 2018-08-08

**Authors:** Michael G. Browne, Jason R. Franz

**Affiliations:** Joint Department of Biomedical Engineering, University of North Carolina at Chapel Hill and North Carolina State University, Chapel Hill, NC, United States of America; University of Colorado Boulder, UNITED STATES

## Abstract

**Introduction:**

Compared to young adults, older adults walk with smaller propulsive forces and a redistribution to more proximal leg muscles for power generation during push-off. Despite this deficit in propulsive function, older adults can increase push-off intensity when encouraged to via real-time biofeedback. However, the specific joint-level modifications used by older adults to enhance propulsive force generation has yet to be elucidated. The purpose of this study was to identify the joint-level modifications used by young and older adults to modulate propulsive forces when walking at their preferred speed.

**Methods:**

9 young and 16 older adults walked at their preferred speed while visual biofeedback prompted them to modulate their propulsive forces using targets prescribed at ±10% and ±20% of their preferred value. Older adults were then divided into groups exhibiting relatively larger or smaller baseline redistribution to more proximal leg muscles for power generation.

**Results:**

Neither young nor either older adult cohort modulated propulsive forces by altering their peak ankle power generation. Instead, subjects increased trailing limb extension and attenuated mechanical power demands at the hip during push-off. Older adults that had a larger baseline redistribution exhibited larger responses to enhanced push-off intensity than their peers–for example, walking with 11% less hip flexor power and 10% more trailing limb extension during push-off when exerting larger than preferred propulsive forces.

**Conclusion:**

Propulsive force biofeedback that elicits larger than preferred propulsive forces also increases trailing limb extension and attenuates mechanical power demands at the hip in older adults most exhibiting a distal-to-proximal redistribution. Our results suggest that considering baseline redistribution may be important in the personalized prescription of interventions aimed at enhancing walking performance by improving push-off intensity.

## Introduction

Older adults (age>65 years) tend to walk at slower speeds with shorter steps and higher metabolic energy costs than young adults [1–4]. Often preceding, and likely contributing to these age-associated changes is a prominent decrease in push-off intensity generated predominantly from the ankle plantarflexor muscles [5–8]. As a potential compensation for this decrease in ankle power, older adults rely more than young adults on hip musculature for power generation, a phenomenon known as a distal-to-proximal redistribution [5]. This redistribution may explain, at least in part, the greater metabolic energy costs of older adults and could thereby be considered maladaptive; the longer muscle fascicles and relatively short tendons spanning the hip are less metabolically favorable than the short fascicles and long, series elastic tendons spanning the ankle [9, 10]. Moreover, increases in proximal leg muscle power generation appear unable to completely offset age-related reductions in ankle power output, as older adults continue to exert smaller propulsive forces (the peak anterior component of the ground reaction force during push-off [F_P_]) than young adults walking at the same speed [11].

Despite reductions in ankle power output and F_P_ compared to young adults, many older adults retain the ability to increase both during walking when faced with task requirements that challenge propulsion. For example, older adults increase ankle power output by up to 24% to walk at speeds faster than preferred [12] and increase F_P_ by approximately 40% to walk uphill at a 9° grade [11]. Based on the apparent availability of these “propulsive reserves” in older adults, our previous work has also demonstrated that visual biofeedback can encourage older adults to increase their F_P_ by up to 25% when walking on level ground at their preferred speed. More curiously, older subjects in that study retained the ability to exert even larger propulsive forces than those exerted normally by young adults [13]. Those subjects simultaneously exhibited increased plantarflexor muscle activation during push-off, alluding to potential neuromechanical improvements at the ankle (i.e. moment and/or power). Still, the specific joint-level modifications used by older adults to enhance their F_P_ generation has yet to be elucidated–leaving a major gap in our understanding.

There are several different means by which older adults could modulate F_P_ in response to biofeedback and, due to known age-related neuromuscular changes, these joint-level modifications could differ from those in young subjects. Evidence from Hsiao et al. (2015) suggests that larger F_P_ during walking can be exerted through a combination of increased trailing limb extension and increased ankle mechanical output during push off, at least to increase walking speed [14–16]. However, other evidence, including studies in older adults, is less equivocal. For example, older adults that increase their walking speed, and thus presumably F_P_, after resistance training do so by increasing ankle power generation in one study [17] but, conversely, by exacerbating their distal-to-proximal redistribution in another [18]. Further confounding our predictions, we recently showed in young adults that the joint-level modifications used to change walking speed can be fundamentally different from those to modulate F_P_ when walking at a constant speed [19]. Can older adults enhance F_P_ generation during walking through increased ankle power output, a potentially favorable response that could mitigate compensatory mechanical power demands at the hip? Alternatively, are older adults functionally limited in their ability to increase ankle power output during walking, a constraint that would require exacerbating their reliance on more proximal leg muscles? Understanding the joint-level mechanisms by which older adults enhance F_P_ generation during walking is important for informing the development and prescription of interventions aimed at improving walking performance in the elderly.

The purpose of this study was to identify the joint-level modifications used by young and older adults to modulate F_P_ when walking at their preferred speed. We first hypothesized that young and older adults would increase/decrease F_P_ by increasing/decreasing ankle power. Second, based on compensatory trade-offs between the ankle and hip musculature, we hypothesized that such changes in F_P_ would be met by changes in hip power that oppose those in ankle power. This outcome would imply that increased F_P_ may reverse the distal-to-proximal redistribution in older subjects while alternatively that decreases in F_P_ would do the opposite, increasing the reliance on hip musculature. Finally, we hypothesized that baseline distal-to-proximal redistribution (i.e. the amount an older adult relies on hip versus ankle musculature for mechanical power generation) would influence the means by which they increase push-off intensity. Specifically, older adults with a greater baseline distal-to-proximal redistribution may be more inclined to exacerbate that redistribution when increasing F_P_.

## Materials and methods

### 2.1. Participants

An *a priori* power analysis determined that n = 8 subjects per group would have 90% power to detect (p<0.05) a 3%BW difference in peak propulsive force between young and older subjects (i.e., 23.0%BW vs. 20.2%BW) [13]. Thus, we recruited 9 healthy young adults (5F/4M) with mean (standard deviation) age: 25.1 (5.6) years, height: 1.76 (0.06) m, and mass: 72.0 (7.1) kg and, to address a subgroup analysis described in more detail below, 16 older adults (11F/5M) with age: 75.3 (3.5) years, height: 1.68 (0.09) m, and mass: 65.0 (11.5) kg to participate. Our exclusion criteria, assessed via health questionnaire, were BMI≥30, self-reported inability to walk comfortably for 20 consecutive minutes, lower extremity fracture within 6 months, neurological disorder affecting the legs, pain during walking, medication causing dizziness, leg prosthesis, and requiring an assistive aid for ambulation. All subjects provided written, informed consent according to a study protocol approved by the University of North Carolina Biomedical Sciences Institutional Review Board.

### 2.2. Procedures

**Fig 1:**
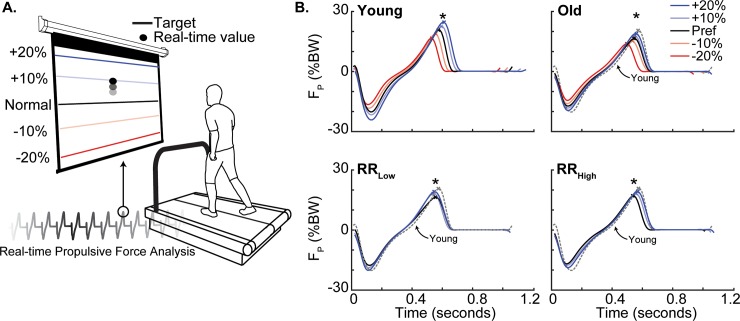
**A.)** Experimental design using real-time peak propulsive force measurements projected in front of participants with targets representing ±10 and 20% different from preferred. **B.)** Group average anterior-posterior ground reaction force by condition across time for all young (top left), all old (top right), RR_Low_ (bottom left), and RR_High_ (bottom right). Dashed grey lines represent young preferred walking as reference. An X on each curve provides a reference for the timing of peak F_P_. Asterisks (*) represent a significant main effect (p<0.05) of F_P_ biofeedback on peak propulsive force.

We first calculated preferred walking speed (PWS) using the average time from 3 repetitions for subjects to walk the middle 3 m of a 10 m walkway using a photo cell timing system (Bower Timing Systems, Draper, UT, USA). Subjects then walked on a dual belt, force-sensing treadmill (Bertec, Columbus, OH, USA) at their PWS for 60 s while a custom Matlab (Mathworks, Natick, MA, USA) script extracted their peak propulsive force (F_P_) from each step, resulting in an average preferred F_P_. While subjects walked, the scripts monitored bilateral ground reaction forces (GRF) at 1000 Hz, detected heel-strike using a 10 N vertical GRF threshold, and then extracted the peak F_P_ value from each step. Then F_P_ biofeedback trials used a second Matlab script which, in real-time, computed a moving average of bilateral peak F_P_ from each set of four consecutive steps and projected those values as a dot on a screen positioned in front of the subject (Fig 1A). We first provided subjects with a biofeedback exploration trial of up to 3 min for them to familiarize themselves with modulating F_P_. To elicit naturally emerging biomechanical patterns associated with the modulation of F_P_, we explained the timing of push-off and that if they pushed harder or softer with their foot during push-off, the dot would rise or fall, respectively. Finally, subjects walked at their PWS for 90 s each while matching their instantaneous F_P_ to targets representing ±10% and ±20% different from preferred, presented in fully-randomized order.

### 2.3. Measurement and analysis

In addition to the exploration trial, we also allowed each subject 30 seconds to accommodate to each condition by using the final 60 seconds for all analyses. Pelvis and lower extremity kinematics were recorded using a 14-camera motion capture system (Motion Analysis Corporation, Santa Rosa, CA, USA) operating at 100 Hz. We used 14 anatomical markers placed bilaterally on subjects’ first and fifth metatarsal heads, calcaneus, lateral malleoli, lateral knee joint center (placed between the tibial and femoral epicondyles), anterior superior iliac spines, and posterior superior iliac spines. An additional 14 tracking markers were attached bilaterally using asymmetric rigid clusters to subjects’ shank and thigh segments. These 28 markers were used during all walking trials and medial knee and ankle markers were added during a static standing trial.

We filtered marker trajectories and GRFs using 4^th^ order low-pass Butterworth filters with cutoff frequencies of 6 Hz and 100 Hz, respectively. We used a static calibration to scale seven segment, 18 degree-of-freedom models of the pelvis and right and left legs [20] using a leg circumduction task to estimate functional hip joint centers [21]. We estimated bilateral sagittal plane hip, knee, and ankle joint angles, moments, and powers using an inverse dynamics routine implementing the filtered marker and force data as described in more detail previously [22]. From each subject’s stride-average curves, we extracted discrete values representing important kinematic and kinetic events. Peaks included F_P_, ankle plantarflexion, hip extension, ankle moment, ankle power, hip power during early stance (H1), and hip power during push-off (H3). We also estimated the positive ankle angular impulse by calculating the area under the positive portion of the ankle moment versus time curve. GRFs were normalized to body weight and reported as a percent and all joint kinetic outcomes were normalized to body mass. We calculated stride length and trailing limb extension at peak F_P_ according to previously published methods [15]. Finally, we took the average between left and right legs for each outcome variable for statistical analysis.

### 2.4. Redistribution Ratio

We divided our older adult subjects into two cohorts: one with a less exaggerated and one with a more exaggerated distal-to-proximal redistribution. Here, we developed the Redistribution Ratio (RR) which uses stance phase positive ankle and hip joint work to operationally define subjects’ reliance on distal vs. proximal leg muscles in generating positive power during walking. The RR is defined according to Eq 1:

$$RR=1-\frac{W_{A}^{+}-W_{H}^{+}}{W_{A}^{+}+W_{H}^{+}}$$ (1)

where $W_{A}^{+}$ refers to the total positive work performed by ankle musculature and $W_{H}^{+}$ refers to total positive work performed by hip musculature. Joint work was calculated using the time integral of the stride-averaged power versus time profile. RR is bounded between 0 and 2, where 0 signifies that all positive work was performed about the ankle and 2 signifies that all positive work is performed about the hip. Accordingly, lower and higher RR values denote low and high distal-to-proximal redistribution, respectively. Specifically, we identified the 8 (50%) lower RR subjects (6F/2M) as “RR_Low_” and the 8 higher RR subjects (5F/3M) as “RR_High_”.

### 2.5. Statistical analysis

We first confirmed that all outcome measures were normally distributed using Shapiro-Wilks tests. We then used independent t-tests between older and young adults to test for significant differences in PWS, F_P_, peak ankle moment, positive ankle angular impulse, peak ankle power, and H1 and H3 peak hip powers. Two planned one-way repeated measures analyses of variance (rmANOVA) then tested for main effects of F_P_ biofeedback on F_P_ and ankle and hip joint kinematics and kinetics for older and young adults. When a significant main effect was found, we focused post-hoc pairwise comparisons between preferred walking and each F_P_ target condition (i.e., ±10%, ±20%). We tested our hypothesis that older adults with a greater baseline distal-to-proximal redistribution may be more inclined to exacerbate that distribution when increasing F_P_ using a two-way mixed factorial ANOVA to test for main effects of and interactions between RR and F_P_ target conditions on ankle and hip joint kinematics and kinetics as well as the RR. An independent t-test, including PWS as a covariate, compared RR between groups while additional independent t-tests compared age, height, mass, and PWS between groups (Young, RR_Low_, RR_High_).

## Results

### 3.1. Joint-level modifications used by young and older subjects to modulate F_P_

**Fig 2:**
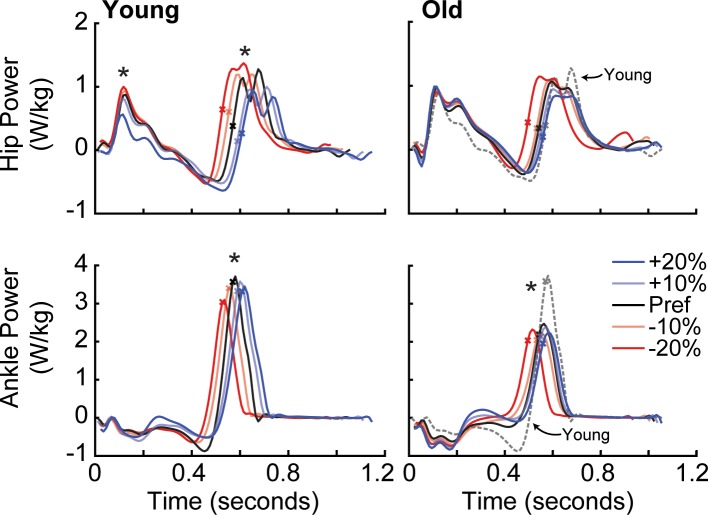
Group average hip and ankle joint powers by condition across time for all young (left) and all older adult subjects (right). Dashed grey lines represent young preferred walking as reference. An X on each curve provides a reference for the timing of peak F_P_. Asterisks (*) represent a significant main effect (p<0.05) of F_P_ biofeedback on peak hip power during early stance (i.e., H1) and push-off (i.e., H3) and on peak ankle power.

Preferred walking speed, and thus the treadmill speed, did not significantly differ between young, 1.30 (0.12) m/s, and older subjects, 1.25 (0.20) m/s (p = 0.498). Compared to young adults, older adults walked habitually at their preferred speed with 19% smaller F_P_ (p = 0.014, Fig 1B), 15% smaller peak ankle moment (p<0.001), 28% smaller peak ankle power (p = 0.002), and a 29% higher RR indicative of a distal-to-proximal redistribution (Fig 2 and Table 1). Older (young) adults successfully modified their F_P_ to respective targets, by an average of -14.6% (-20.6%) and +14.1% (+20.3%) when targeting changes of -20% and +20%, respectively (Fig 1B and Table 1).

**Table 1:** Peak kinematic and kinetic outcome measures for all groups and conditions.

		Fp Biofeedback Target					Main Effect
		-20%	-10%	Preferred	+10%	+20%	p-value
**Peak Fp** **(% Body Weight)**	**Young**	16.7 ± 1.3*	18.9 ± 1.5*	21.1 ± 1.8	22.8 ± 1.8*	25.3 ± 2.2*	<0.001
	**Old**	14.6 ± 2.9*	16.0 ± 3.3*	17.1 ± 4.3	18.5 ± 4.3*	19.5 ± 4.7*	<0.001
	**RR**_**Low**_	—	—	16.9 ± 3.7	18.6 ± 3.7*	19.6 ± 4.0*	<0.001
	**RR**_**High**_	—	—	17.2 ± 5.0	18.4 ± 5.1*	19.4 ± 5.6*	<0.001
**Peak Ankle Plantarflexion (°)**	**Young**	-13.9 ± 5.1*	-18.6 ± 4.9*	-23.0 ± 7.6	-28.5 ± 7.7*	-30.2 ± 7.8*	<0.001
	**Old**	-14.1 ± 8.0*	-16.5 ± 8.5	-18.9 ± 8.0	-20.5 ± 8.8*	-20.6 ± 8.2*	<0.001
	**RR**_**Low**_	—	—	-20.5 ± 8.1	-22.4 ± 8.2	-22.2 ± 7.5	NS
	**RR**_**High**_	—	—	-17.3 ± 8.2	-18.6 ± 9.5	-19.1 ± 9.0	NS
**Peak Hip Extension (°)**	**Young**	-8.1 ± 6.0*	-10.1 ± 6.7	-12.3 ± 4.6	-15.0 ± 4.9*	-15.5 ± 4.7*	<0.001
	**Old**	0.0 ± 6.6*	-2.6 ± 6.8*	-5.7 ± 7.2	-4.8 ± 7.2	-5.5 ± 7.3	<0.001
	**RR**_**Low**_	—	—	-6.5 ± 8.4	-4.9 ± 9.1	-5.9 ± 9.0	NS
	**RR**_**High**_	—	—	-4.9 ± 6.2	-4.6 ± 5.2	-5.1 ± 5.7	NS
**Trailing Limb Extension (°)**	**Young**	14.6 ± 1.3*	15.1 ± 3.0	16.7 ± 1.6	18.0 ± 1.5*	18.7 ± 1.3*	<0.001
	**Old**	14.4 ± 2.7*	15.7 ± 2.6	16.3 ± 2.9	17.0 ± 2.7*	17.4 ± 3.0*	<0.001
	**RR**_**Low**_	—	—	16.8 ± 2.6	17.4 ± 2.5	17.7 ± 2.9	NS
	**RR**_**High**_	—	—	15.7 ± 3.3	16.6 ± 3.1*	17.2 ± 3.2*	<0.001
**Stride Length (m)**	**Young**	1.24 ± 0.09*	1.30 ± 0.10	1.34 ± 0.05	1.41 ± 0.09*	1.46 ± 0.11*	<0.001
	**Old**	1.10 ± 0.14*	1.19 ± 0.15	1.21 ± 0.15	1.22 ± 0.12	1.24 ± 0.13	<0.001
	**RR**_**Low**_	—	—	1.23 ± 0.19	1.21 ± 0.16	1.23 ± 0.15	NS
	**RR**_**High**_	—	—	1.19 ± 0.10	1.23 ± 0.09	1.26 ± 0.12	NS
**Peak Ankle Moment (Nm/kg)**	**Young**	-1.45 ± 0.12*	-1.54 ± 0.17*	-1.60 ± 0.17	-1.59 ± 0.18	-1.54 ± 0.18	<0.001
	**Old**	-1.27 ± 0.12*	-1.29 ± 0.11*	-1.36 ± 0.13	-1.35 ± 0.14	-1.31 ± 0.15	0.008
	**RR**_**Low**_	—	—	-1.41 ± 0.12	-1.39 ± 0.16	-1.32 ± 0.17	NS
	**RR**_**High**_	—	—	-1.32 ± 0.13	-1.31 ± 0.12	-1.30 ± 0.14	NS
**Ankle Angular Impulse (Nms/kg)**	**Young**	0.38 ± 0.05*	0.43 ± 0.05	0.44 ± 0.03	0.51 ± 0.04*	0.51 ± 0.07*	<0.001
	**Old**	0.40 ± 0.07	0.43 ± 0.07	0.43 ± 0.07	0.49 ± 0.09*	0.50 ± 0.09*	<0.001
	**RR**_**Low**_	—	—	0.44 ± 0.06	0.50 ± 0.09*	0.51 ± 0.09*	<0.001
	**RR**_**High**_	—	—	0.43 ± 0.07	0.48 ± 0.09*	0.49 ± 0.09*	<0.001
**Peak Ankle Power** **(W/kg)**	**Young**	3.25 ± 0.78*	3.52 ± 0.87*	3.85 ± 0.85	3.79 ± 0.79	3.72 ± 0.89	0.001
	**Old**	2.68 ± 0.68	2.56 ± 0.62*	2.79 ± 0.66	2.68 ± 0.61	2.54 ± 0.61*	0.037
	**RR**_**Low**_	—	—	2.78 ± 0.74	2.72 ± 0.65	2.46 ± 0.52	NS
	**RR**_**High**_	—	—	2.80 ± 0.61	2.65 ± 0.62	2.62 ± 0.71	NS
**H1 Hip Power Peak** **(W/kg)**	**Young**	1.09 ± 0.37	1.04 ± 0.35	0.98 ± 0.42	0.91 ± 0.34	0.71 ± 0.21	0.007
	**Old**	1.03 ± 0.25	1.08 ± 0.29	1.05 ± 0.28	1.10 ± 0.34	1.11 ± 0.35	NS
	**RR**_**Low**_	—	—	0.94 ± 0.16	1.00 ± 0.25	0.99 ± 0.21	NS
	**RR**_**High**_	—	—	1.17 ± 0.34	1.21 ± 0.4	1.23 ± 0.43	NS
**H3 Hip Power Peak** **(W/kg)**	**Young**	1.59 ± 0.30	1.55 ± 0.27	1.62 ± 0.43	1.40 ± 0.30*	1.28 ± 0.31*	<0.001
	**Old**	1.45 ± 0.42	1.45 ± 0.45	1.44 ± 0.49	1.35 ± 0.40	1.28 ± 0.43	NS^a^
	**RR**_**Low**_	—	—	1.30 ± 0.36	1.29 ± 0.28	1.15 ± 0.21	NS
	**RR**_**High**_	—	—	1.58 ± 0.58	1.41 ± 0.51*	1.41 ± 0.55	0.033
**Redistribution Ratio**	**Young**	0.95 ± 0.15*	0.86 ± 0.17*	0.75 ± 0.16	0.66 ± 0.11	0.57 ± 0.13*	<0.001
	**Old**	1.04 ± 0.17*	1.01 ± 0.15	0.96 ± 0.16	0.94 ± 0.20	0.94 ± 0.18	0.013
	**RR**_**Low**_	—	—	0.84 ± 0.08	0.82 ± 0.11	0.85 ± 0.08	NS
	**RR**_**High**_	—	—	1.08 ± 0.13	1.07 ± 0.18	1.03 ± 0.21	NS

*significant pairwise difference from Preferred (p<0.05); NS: not significant (p>0.05); ^a^ statistical trend (p = 0.078)

We observed significant main effects of modified F_P_ on peak ankle moment (p<0.001) and power generation (p<0.001) in both older and young adults (Fig 2). Pairwise comparisons revealed that these effects were almost exclusively significant for decreases when walking with smaller than preferred F_P_. Older adults, however, also demonstrated decreased ankle power generation when targeting 20% larger than preferred F_P_. We also observed significant main effects of modified F_P_ on ankle angular impulse in both young and older adults (p<0.001). Here, pairwise comparisons revealed that these effects were significant primarily for increases when walking with larger than preferred F_P_ (Table 1). Both groups tended to rely less on positive hip power generation during push-off (i.e., H3 peak) across the range of F_P_ targets, from -20% to +20% (main effects, old: p = 0.078, young: p<0.001). These effects were most prevalent for increases in F_P_, with older (young) adults decreasing hip flexor power by 11% (21%) when targeting 20% larger than preferred F_P_.

**Fig 3:**
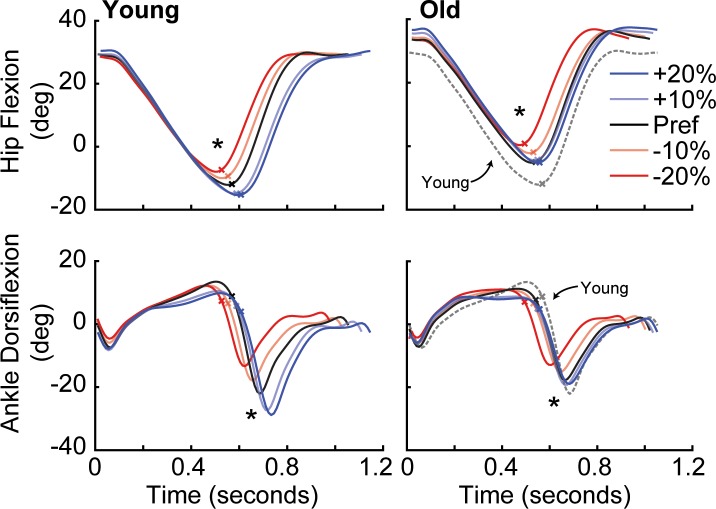
Group average hip and ankle joint angles by condition across time for all young (left) and all older adult subjects (right). Positive values represent flexion. Dashed grey lines represent young preferred walking as reference. An X on each curve provides a reference for the timing of peak F_P_. Asterisks (*) represent a significant main effect (p<0.05) of F_P_ biofeedback on peak joint extension.

Subjects also altered their hip and ankle joint kinematics when modulating F_P_. Specifically, we found for both age groups significant main effects of F_P_ target condition on trailing limb extension, peak ankle and hip extension, and stride length (p≤0.001) (Fig 3 and Table 1). Pairwise comparisons in older (young) adults showed that changes in trailing limb extension mirrored those in F_P_, decreasing by 11.6% (12.6%) when targeting 20% smaller than preferred F_P_, and increasing by 7.2% (12.1%) when targeting 20% larger than preferred F_P_ in older (young) subjects. However, the pairwise response for other outcome measures differed between groups. Compared to preferred walking, young adults decreased peak ankle and hip extension and walked with shorter strides when targeting smaller F_P_ and increased each when targeting larger F_P_ (p<0.001). In contrast, we found significant pairwise comparisons in older adults for decreases in ankle plantarflexion, peak hip extension, and shorter stride length when targeting smaller than preferred F_P_ (p<0.033); only ankle plantarflexion increased significantly when older adults targeted larger than preferred F_P_ (p<0.047).

### 3.2. Effects of baseline distal-to-proximal redistribution in older adults

**Fig 4:**
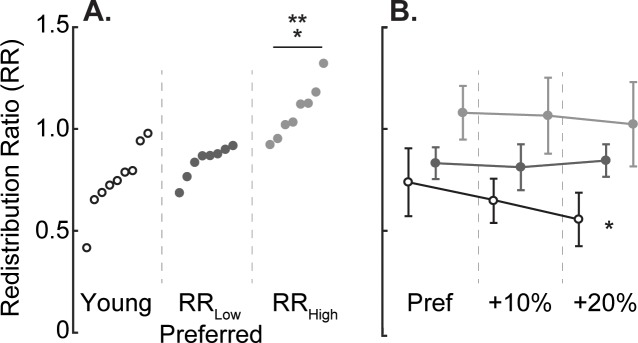
**A.)** Individual subject Redistribution Ratio (RR) value for Young, RR_Low_, and RR_High_. Single asterisks (*) represent a significant pairwise difference between RR_High_ and Young. Double asterisks (**) represent a significant pairwise difference between RR_High_ and RR_Low,_ including preferred walking speed as a covariate **B.)** Group average and standard deviation Redistribution Ratio by increased propulsive force for Young (open circles), RR_Low_ (dark grey circles), and RR_High_ (light grey circles). Asterisks (*) represent a significant main effect (p<0.05) of increased F_P_ biofeedback on the Redistribution Ratio.

RR was indistinguishable between RR_Low_ subjects and young adults (mean [sd]: 0.84 [0.08] vs. 0.75 [0.16], p = 0.168), but was significantly higher in RR_High_ subjects (1.08 [0.20], p<0.001 vs. Young, p<0.001 vs RR_Low_; Fig 4 and Table 1). RR_Low_ and RR_High_ did not significantly differ in any outcome measure during preferred walking, including PWS. In addition, both groups of older subjects were relatively successful using F_P_ biofeedback, increasing by 15.5% (RR_Low_) and 12.7% (RR_High_) when targeting 20% larger than preferred F_P_ (Fig 1B).

**Fig 5:**
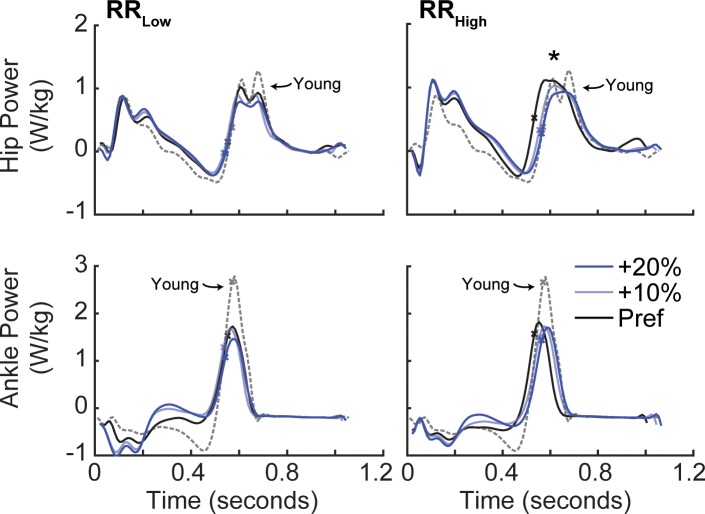
Group average hip and ankle joint powers by condition across time for RR_Low_ (left) and RR_High_ (right). Dashed grey lines represent young preferred walking as reference. An X on each curve provides a reference for the timing of peak F_P_. Asterisks (*) represent a significant main effect (p<0.05) of F_P_ biofeedback on peak hip power during early stance (i.e., H1) and push-off (i.e., H3) and on peak ankle power.

Neither RR_Low_ nor RR_High_ subjects increased F_P_ by adjusting peak ankle power (Fig 5). Both groups did increase F_P_, however, by adjusting positive ankle angular impulse by over 14% when targeting 20% larger than preferred F_P_ (p<0.001). When targeting larger than preferred F_P_, for example in the +10% condition, RR_High_ but not RR_Low_ decreased hip flexor power generation during push-off by 11% (p = 0.047). In addition, only RR_High_ subjects increased trailing limb extension when targeting larger than preferred F_P_ (p<0.001). Conversely, RR_Low_ subjects did not modulate hip power nor any kinematic outcome measure when targeting larger than preferred F_P._

## Discussion

Older adults walk with a distal-to-proximal redistribution (i.e. decreased power generated by ankle musculature in favor of increased power generated by hip musculature) and reduced propulsive forces (F_P_) during push-off compared to young adults. Despite these age-related changes, older adults can increase F_P_ volitionally when prompted via visual biofeedback [13]. We used a similar visual biofeedback paradigm to uncover the joint-level modifications used by young and older subjects to modulate F_P_ during walking. Our intuition led us to anticipate that older subjects would increase F_P_ when walking at their preferred speed either through: (*i*) increased ankle power generation and a reversal of their distal-to-proximal redistribution, or (*ii*) further exacerbating their distal-to-proximal redistribution (i.e. relying even more on hip musculature for power generation). Our results reveal an interesting third option; older adults opt to modulate F_P_ without any change to ankle power generation–a nearly identical response to young adults completing the same task. Instead, when increasing F_P_, older adults increase trailing limb extension, use a larger ankle angular impulse, and adopt potentially favorable reductions in hip flexor power generation. Our findings also suggest that older adults who use a greater distal-to-proximal redistribution for power generation in walking than their peers exhibit more favorable biomechanical changes at the hip when increasing push-off intensity via biofeedback.

### 4.1. Joint-level modifications used to modulate propulsive forces

Positive power generated about the ankle during the push-off phase of walking contributes both to accelerating the body’s center of mass (CoM) and initiating leg swing [23, 24]. Consistent with previous efforts to improve push-off intensity during walking, neither young nor older adults responded to biofeedback designed to increase F_P_ with any increase in peak ankle power [25]. We caution against interpreting this finding to imply that older adults are incapable of increasing their peak ankle power generation during walking. While the functional consequences of sarcopenia (i.e., the loss of skeletal muscle mass) and muscle weakness are well-documented [26, 27], our results imply only that older adults opt not to alter their peak ankle moment nor power when walking with larger than preferred F_P_. Indeed, because our young adult subjects also failed to increase their peak ankle joint kinetics, our results do not necessarily allude to any specific functionally limiting impairment in elderly gait. Further, the ability of both young and older adults to increase positive ankle angular impulse suggests an increase in cumulative loading of ankle musculature that does not reveal itself in values isolated to the push-off phase of walking. Our findings highlight a clear opportunity for the implementation of biofeedback paradigms that focus specifically on enhancing mechanical power output from the plantarflexor muscles, perhaps through the use of real-time inverse dynamics [28].

We also found that changes in peak trailing limb extension during push-off mirrored those in F_P_, independent of age. Hsiao et.al. (2015) implicated trailing limb extension and peak ankle joint kinetics as responsible for increases in F_P_ that accompanied their subjects walking faster than preferred. In partial agreement with their model predictions, we observed that subjects increased trailing limb extension, but not peak ankle joint kinetics, to walk at their preferred speed with larger F_P_ [15]. This disconnect suggests that increased peak ankle joint kinetics may be unique to increases in walking speed and not a prerequisite for increasing F_P_. Indeed, ankle joint moment and power increase relatively linearly with walking speed [19, 29]. Instead, at a given walking speed, increased trailing limb extension would serve to reorient the GRF vector more anteriorly and thereby produce larger F_P_. In addition, if this change in limb posture were to permit the GRF vector to pass closer to the hip joint during push-off, this may explain the smaller hip flexor power requirements when walking with larger than preferred F_P_ that we discuss in more detail below.

### 4.2. The effects of baseline distal-to-proximal redistribution

As a second major goal of this study, we sought to determine whether baseline distal-to-proximal redistribution (i.e. the amount to which an older adult relies on hip versus ankle musculature for mechanical power generation) would influence how older adults increase their push-off intensity. Here, we introduce the redistribution ratio, a metric for quantifying the extent to which an individual walks with a distal-to-proximal redistribution calculated using the total positive ankle and hip joint work performed across the stance phase. Indeed, our older adult subjects walked with a wide spectrum of baseline redistribution (Fig 4) that may be relevant to understanding their response to interventions designed to enhance push-off intensity. RR_Low_ subjects successfully increased F_P_ to values larger than preferred, but apparently did so by only increasing ankle angular impulse. In contrast, RR_High_, who walked with a more exaggerated distal-to-proximal redistribution than their peers, exhibited larger and more pervasive responses. We believe the joint-level modifications used by RR_High_ to increase F_P_ may suggest interesting functional implications for individuals with a higher baseline distal-to-proximal redistribution.

One of the most interesting changes evident in RR_High_ but not RR_Low_ was that hip flexor power generation during push-off scaled inversely with F_P_; walking with larger than preferred F_P_ decreased hip flexor power generation. That a reduction in positive power generation would accompany larger F_P_ is at first counterintuitive. However, hip flexor power generation serves to pull the trailing leg into swing through concentric action of hip flexor muscles (e.g., iliacus, psoas, and rectus femoris). Consistent with the premise of Siegel et.al. (2004), excessive hip flexor power generation to initiate leg swing may simultaneously inhibit ankle power generation from directly contributing to F_P_ production during push-off [30]. Lewis and Ferris (2008) observed a similar phenomenon of decreased hip power generation when their young adult subjects tried to walk with an exaggerated ankle strategy [25]. Indeed, attenuating excessive hip flexor power generation may have facilitated older adults getting ‘more bang for their ankle power buck’. Moreover, at least in health young adults, leg swing initiation ordinarily accounts for as much as 10% of the metabolic cost of walking–a value that, due to their reliance on less economically favorable leg muscles for power generation, could be much higher in older adults [31]. Accordingly, we posit that attenuating excessive mechanical power demands at the hip may ultimately have metabolic benefits–a logical next step in this line of research.

### 4.3. Age-related biomechanical plasticity?

In their seminal paper, DeVita and Hortobagyi (2000) coined the term “biomechanical plasticity” to describe the age-associated shift in mechanical power generation from muscles spanning the ankle to muscles spanning the hip during walking compared to young adults [5]. However, plasticity is a term derived from mechanics of materials that refers to permanent deformation–an irreparable change in the mechanical state of a body that has yielded to external forces. Therefore, in the strictest of interpretations, biomechanical plasticity thus implies that the proximal shift in joint power generation in older adults having yielded to age-related neuromuscular changes may prove permanent. However, we have shown here that encouraging older adults to normalize their F_P_ to values seen in young adults may attenuate compensatory mechanical power demands on proximal leg muscles. Interestingly, these changes were only evident in RR_High_ subjects (i.e., older subjects we would interpret as using more of a distal-to-proximal redistribution during preferred walking than their peers) and young adults (i.e. subjects we would interpret as having normative patterns of joint power generation). Accordingly, despite relatively invariant ankle power generation during push-off, observed here for older and young subjects, we posit that certain components of age-related biomechanical plasticity may in fact be more elastic than previously appreciated. In this context, older adults may not have permanently yielded to age-related neuromuscular changes. Rather, their greater hip power generation during walking, considered by some maladaptive for its potential impact on walking economy, can, under the appropriate circumstances, return to its original, more youthful, state. Unfortunately, these results do little to address why older adults instinctively opt to walk at their preferred speed with smaller peak F_P_ and greater hip power generation than young adults. Nevertheless, that some older adults retain the capacity to walk with more youthful biomechanical patterns suggests that some may walk with a certain biomechanical *elasticity* and may be trained to walk with more favorable patterns of joint power generation. Taken together, our results suggest that considering baseline patterns of joint power generation (e.g. distal-to-proximal redistribution) may be an important step toward the more personalized prescription of interventions aimed at enhancing walking performance by improving push-off intensity. Future work will investigate clinically viable surrogates for F_P_ using wearable sensors such as inertial measurement units.

### 4.4. Limitations

We acknowledge several limitations of this study. First, our older subjects were healthy and physically active and thus may not represent those we would consider at risk for age-related mobility impairments. Nevertheless, these older adults walked with many of the hallmark and, in our opinion, unfavorable biomechanical changes attributed to elderly gait—deficits in F_P_ and ankle power generation compared to young adults. In addition to providing subjects time to practice walking with visual biofeedback, we analyzed the last 60 s of each walking trial to allow more time for subjects to acclimate to each biofeedback target. However, it is possible that subjects had not grown fully accustomed to each target. Indeed, while outside the scope of this study, learning and adaptation to propulsive biofeedback remains an interesting and important future direction. Our participants walked on the treadmill at their overground PWS, and there may be differences in how individuals respond to walking and/or biofeedback on a treadmill compared to overground. We also excluded the knee in our joint-level kinematic and kinetic analysis. Net positive work performed by muscles spanning the knee is relatively small in walking and, instead, is more prominent in performing negative work during the stance phase of walking [32].

## Conclusion

In summary, we investigated the joint-level modifications used by young and older adults to modulate propulsive forces during walking using a novel visual biofeedback paradigm. Surprisingly, modulating propulsive forces does not require changes in the mechanical output of the plantarflexor muscles. Instead, walking with larger than preferred propulsive forces are instinctively accommodated during push-off by increasing trailing limb extension and attenuating mechanical power demands at the hip–potentially favorable changes in elderly gait that we also find are most prominent in older adults who use more of a distal-to-proximal redistribution for power generation in walking than their peers.

## Supporting information

S1 File Supplementary data. (XLSX)
